# Defining functional diversity for lignocellulose degradation in a microbial community using multi-omics studies

**DOI:** 10.1186/s13068-018-1164-2

**Published:** 2018-06-18

**Authors:** Anna M. Alessi, Susannah M. Bird, Nicola C. Oates, Yi Li, Adam A. Dowle, Etelvino H. Novotny, Eduardo R. deAzevedo, Joseph P. Bennett, Igor Polikarpov, J. Peter W. Young, Simon J. McQueen-Mason, Neil C. Bruce

**Affiliations:** 1 Department of Biology, Centre for Novel Agricultural Products, University of York, York, YO10 5DD UK; 2 Department of Biology, Bioscience Technology Facility, University of York, York, YO10 5DD UK; 3 Embrapa Solos, Rio de Janeiro, RJ Brazil; 4 Grupo de Biotecnologia Molecular, Instituto de Física de São Carlos, Universidade de São Paulo, São Carlos, SP Brazil; 5 Department of Biology, University of York, York, YO10 5DD UK

**Keywords:** CAZy, Metasecretome, Lignocellulose

## Abstract

**Background:**

Lignocellulose is one of the most abundant forms of fixed carbon in the biosphere. Current industrial approaches to the degradation of lignocellulose employ enzyme mixtures, usually from a single fungal species, which are only effective in hydrolyzing polysaccharides following biomass pre-treatments. While the enzymatic mechanisms of lignocellulose degradation have been characterized in detail in individual microbial species, the microbial communities that efficiently breakdown plant materials in nature are species rich and secrete a myriad of enzymes to perform “community-level” metabolism of lignocellulose. Single-species approaches are, therefore, likely to miss important aspects of lignocellulose degradation that will be central to optimizing commercial processes.

**Results:**

Here, we investigated the microbial degradation of wheat straw in liquid cultures that had been inoculated with wheat straw compost. Samples taken at selected time points were subjected to multi-omics analysis with the aim of identifying new microbial mechanisms for lignocellulose degradation that could be applied in industrial pre-treatment of feedstocks. Phylogenetic composition of the community, based on sequenced bacterial and eukaryotic ribosomal genes, showed a gradual decrease in complexity and diversity over time due to microbial enrichment. Taxonomic affiliation of bacterial species showed dominance of *Bacteroidetes* and *Proteobacteria* and high relative abundance of genera *Asticcacaulis*, *Leadbetterella* and *Truepera*. The eukaryotic members of the community were enriched in peritrich ciliates from genus *Telotrochidium* that thrived in the liquid cultures compared to fungal species that were present in low abundance. A targeted metasecretome approach combined with metatranscriptomics analysis, identified 1127 proteins and showed the presence of numerous carbohydrate-active enzymes extracted from the biomass-bound fractions and from the culture supernatant. This revealed a wide array of hydrolytic cellulases, hemicellulases and carbohydrate-binding modules involved in lignocellulose degradation. The expression of these activities correlated to the changes in the biomass composition observed by FTIR and ssNMR measurements.

**Conclusions:**

A combination of mass spectrometry-based proteomics coupled with metatranscriptomics has enabled the identification of a large number of lignocellulose degrading enzymes that can now be further explored for the development of improved enzyme cocktails for the treatment of plant-based feedstocks. In addition to the expected carbohydrate-active enzymes, our studies reveal a large number of unknown proteins, some of which may play a crucial role in community-based lignocellulose degradation.

**Electronic supplementary material:**

The online version of this article (10.1186/s13068-018-1164-2) contains supplementary material, which is available to authorized users.

## Background

Soil and composting microbiomes are diverse and complex microbial communities that degrade plant cell wall biomass. Globally, these microbiomes make a significant contribution to the release of nutrients and recycling of carbon from this highly abundant yet recalcitrant material [1]; however, due to the complexity and the diversity of species, questions about the roles of community members remain unanswered. Furthermore, an understanding of how microbial communities interact in deconstructing lignocellulose conveys important benefits from an industrial perspective and has the potential to provide sources of biocatalysts for the conversion of agricultural residues into biofuels and commodity chemicals.

Lignocellulose degradation in ecosystems like compost and soil is governed by the synergistic action of oxidative and hydrolytic enzymes that break the linkages within and between cellulose, hemicellulose, and lignin [2]. In order to facilitate this process, a variety of interactions occur between different groups of microorganisms. The community structure depends on many environmental factors such as oxygen content, plant origin, soil residues, temperature, pH, phase of the lignocellulose degradation and chemical nature of the exposed biomass [3]. Additionally, microbial competition, driven by the presence of sugars and other nutrients enzymatically released from the lignocellulose, results in a network of metabolic interactions and dependencies between individual species, rendering many of the species present unculturable in isolation. A comprehensive assessment of the microbial community to degrade lignocellulose, therefore, can only be achieved through the combination of ‘omics techniques [4–6].

Whole-metagenome shotgun sequencing studies have proven invaluable to predict the functional potential of complex microbial communities [7, 8]. The recent development of transcriptome sequencing allows a direct measure of the community function under specific growth conditions [9, 10]. In combination with metaproteomics or metabolomics, metatranscriptomics has the potential to create multi-dimensional reports of how the microbes in communities respond to a dynamic environment. Multiple studies have employed ‘omics approaches to investigate the response of the microbiome to external factors such as dietary [11] or xenobiotic [12, 13] stimuli. The majority of these reports have largely focused on a human GI tract microbiome; and though, an increasing number of studies have been performed on ex vivo ecosystems such as marine [14], soil [4], acid mine drainage [15, 16] and anaerobic systems [17]. To the best of our knowledge, limited attempts have been made to link the microbial diversity, metabolic activities, and respective protein abundances in lignocellulose degrading communities by using a combination of culture-independent approaches [4–6, 18].

In this paper, we evaluate time-driven changes in gene expression with extracellular protein production from a lignocellulose-degrading microbial community, and link these patterns to stages of wheat straw degradation.

## Results

### Dynamics of wheat straw degradation

**Fig. 1:**
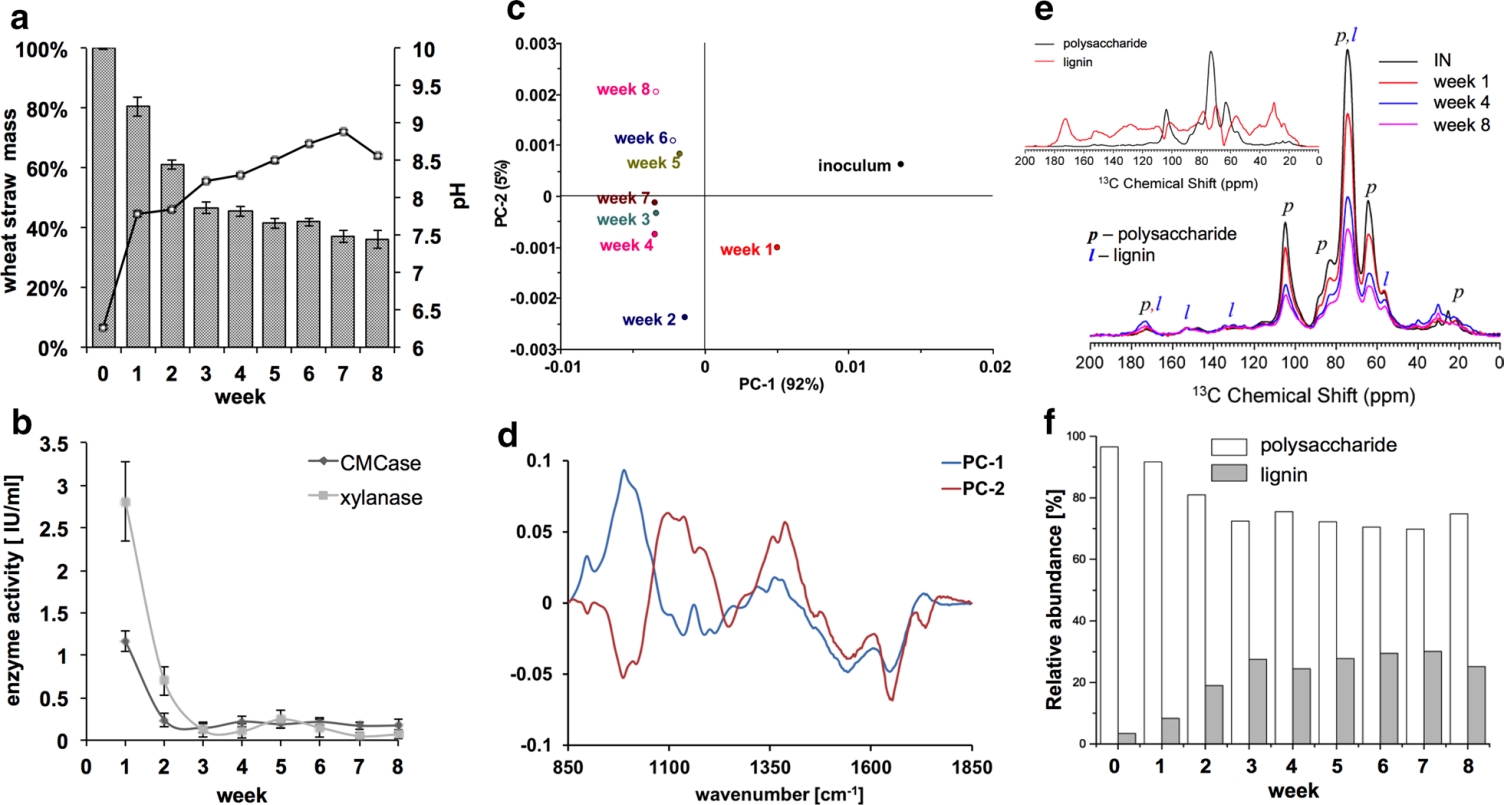
Compositional analysis of wheat straw biomass. **a** The weight of dried wheat straw residues collected from weekly time points of compost-derived cultures and pH of the culture supernatants. **b** Cellulase and xylanase activity in the culture supernatant assayed using carboxymethyl cellulose (CMC) and xylan from beechwood as substrates. Enzyme activity is expressed as μmol of glucose or xylose equivalents released per minute per ml of supernatant (IU/ml). Data in (**a**) and (**b**) represent mean ± SD and *n* = 3, *x*-axis = week. **c** Principal component analysis (PCA) of baseline corrected and peak normalized FTIR spectra in the range of 850–1850 cm^−1^, *n* = 9. **d** Loading plots of PC-1 and PC-2 from the PCA in (**c**). **e** ^13^C ssNMR spectra of untreated and degraded wheat straw indicating qualitative differences in the lignocellulosic fraction between analysed time points. Inset figure shows results of the MCR analysis of the ^13^C ssNMR data on the spectral variation that can be separated into two components associated with polysaccharides and lignin. **f** Relative abundance of polysaccharide and lignin components in wheat straw samples collected from weekly time points of compost-inoculated cultures, *x*-axis = week

Wheat straw was selected as the sole carbon source for shake flask cultures which were inoculated with wheat straw compost. Initially, to determine the rate of degradation of this substrate, biomass samples were removed over periods of time and the dry weight was determined. A rapid loss of 53% of the dry weight was observed over the first three weeks, followed by a slower reduction in weight loss over the next five weeks (64% dry mass reduction after 8 weeks) and an increase of pH from 6.2 to 8.5 (Fig. 1a). Significant morphological changes in the wheat straw biomass were also observed which were consistent with the common effects of plant decay, such as reduction of straw particle size, darkening and biomass softening [7, 19] (Additional file 2: Fig. S1).

Dried and milled wheat straw samples were subsequently analysed by FTIR. The principal component analysis (PCA) of FTIR spectra separated the control sample (time point 0) from the culture samples collected in later weeks (PC-1) (Fig. 1c). The PCA also showed the separation between samples from early and later time points (PC-2). The major peak in the loading plot for PC-1 was observed at wavenumber 950–1100 cm^−1^ and corresponds to cell wall polysaccharides (Fig. 1d) [20]; however, the loading plot for PC-2 showed an enhanced area at wavenumber 1350-1390 cm^−1^ and 1090–1180 cm^−1^ that corresponds to deformation of C–H, C–O bonds of cellulose, xylan and lignin [21] (deformation of syringyl and guaiacyl residues) (Fig. 1c).

Further to FTIR analysis, we collected ssNMR spectra for the control and compost-inoculated wheat straw samples (Fig. 1e). Typical polysaccharide and lignin peaks were assigned based on Rezende et al. [22]. A direct visual inspection of the spectra showed that the intensity of signals due to polysaccharides gradually decrease relative to the lignin signals, indicating a decrease in the relative content of polysaccharides in the biomass samples. To provide a more systematic evaluation of the variability in the ssNMR spectra, a PCA followed by a multivariate curve resolution (MCR) procedure was performed. The PCA showed that the variability of the ssNMR spectra among the samples can be explained by two components (A and B), both associated to the main structural polysaccharide and lignin polymers in the biomass. As shown in Fig. 1e, the major signals in the MCR predicted spectra of component A are assigned to polysaccharides: 60–105 ppm from *O*-alkyl of polysaccharides and 21 ppm typical of hemicellulose. The predicted spectra of component B have signals assigned to lignin (for instance at 151, 128, 55 and 30 ppm) and also signals of partially oxidized polysaccharides to glucuronic acids. There is an expected spectral broadening of the polysaccharide signals due to the increase in the heterogeneity of the material produced from the microbial degradation of the lignocellulose. The estimated spectrum corresponds to band broadening with concomitant reduction of the intensity in the central region of the spectrum, which generates an inverted central peak flanked by positive signals. There is a contribution in the carboxyl region (~ 180 ppm), which could be derived from partially oxidized moieties in the sample, including glucuronic acids.

Thus, we denote component A as unaltered polysaccharides and component B as lignin plus partially oxidized polysaccharides. Based on MCR analysis, we calculated the amount of each spectral component in wheat straw untreated and treated samples (Fig. 1f). It was observed that the relative amount of unaltered polysaccharides in the samples linearly decreased by up to ~ 30% after 3-week post-inoculation and remains mostly constant afterwards. The decrease of unaltered polysaccharides results in the relative increase of the component B, i.e. recalcitrant lignin and partially oxidized polysaccharides. The relative increase in the lignin content of the wheat straw samples is supported by the FTIR results.

Consistent with the observed polysaccharide degradation, xylanase and endo-cellulase activities were detected in the culture supernatants. We observed the highest enzymatic activity during the first week of growth (Fig. 1b); however, both xylanase and cellulase activity dropped dramatically to 23–24% of the initial enzyme activity 2-week post-inoculation. Xylanase and endo-cellulase activity dropped to 4.6 and 15.5% of the initial activity in week three samples, respectively. After the third week, post-inoculation, the level of xylanase and endo-cellulase activities in the culture supernatant reached a plateau and no further significant differences were observed, in line with the ssNMR-MCR results.

Based on the data obtained from the biomass analysis, we selected three time points for further study—early, mid and late stages of lignocellulose degradation corresponding to week one, three and six. A combination of various culture-independent approaches was applied in order, firstly, to determine the taxonomic diversity of the culture community by sequence analysis of 16S and 18S rRNA amplicons; secondly, to evaluate gene expression using metatranscriptome (MT) RNA sequencing; and, thirdly, to identify proteins produced by the community at distinct time points by shotgun LC–MS/MS-based metaexoproteomics (MP). Overall, 9.2 M raw reads were generated from amplicon sequencing using an Ion Torrent platform, 327 M raw reads from the RNA-seq using an Illumina HiSeq platform and 0.27 M peptide spectra using a maXis qTOF LC–MS/MS (Additional file 1: Table S1–S3).

### Amplicon sequencing to study culture community structure and dynamics

We assessed the bacterial and eukaryotic community structure of the initial wheat straw compost inoculum and liquid in vitro cultures using amplicon sequencing of 16S and 18S SSU rRNA. From the sequencing of the eukaryotic 18S gene, a total of 3040 operational taxonomic units (OTUs) were identified at 97% identity level across all samples. A greater number of prokaryotic species were observed from the sequencing of the 16S amplicon. In total, 6949 bacterial OTUs were constructed for all sequenced samples (*n* = 11).

**Fig. 2:**
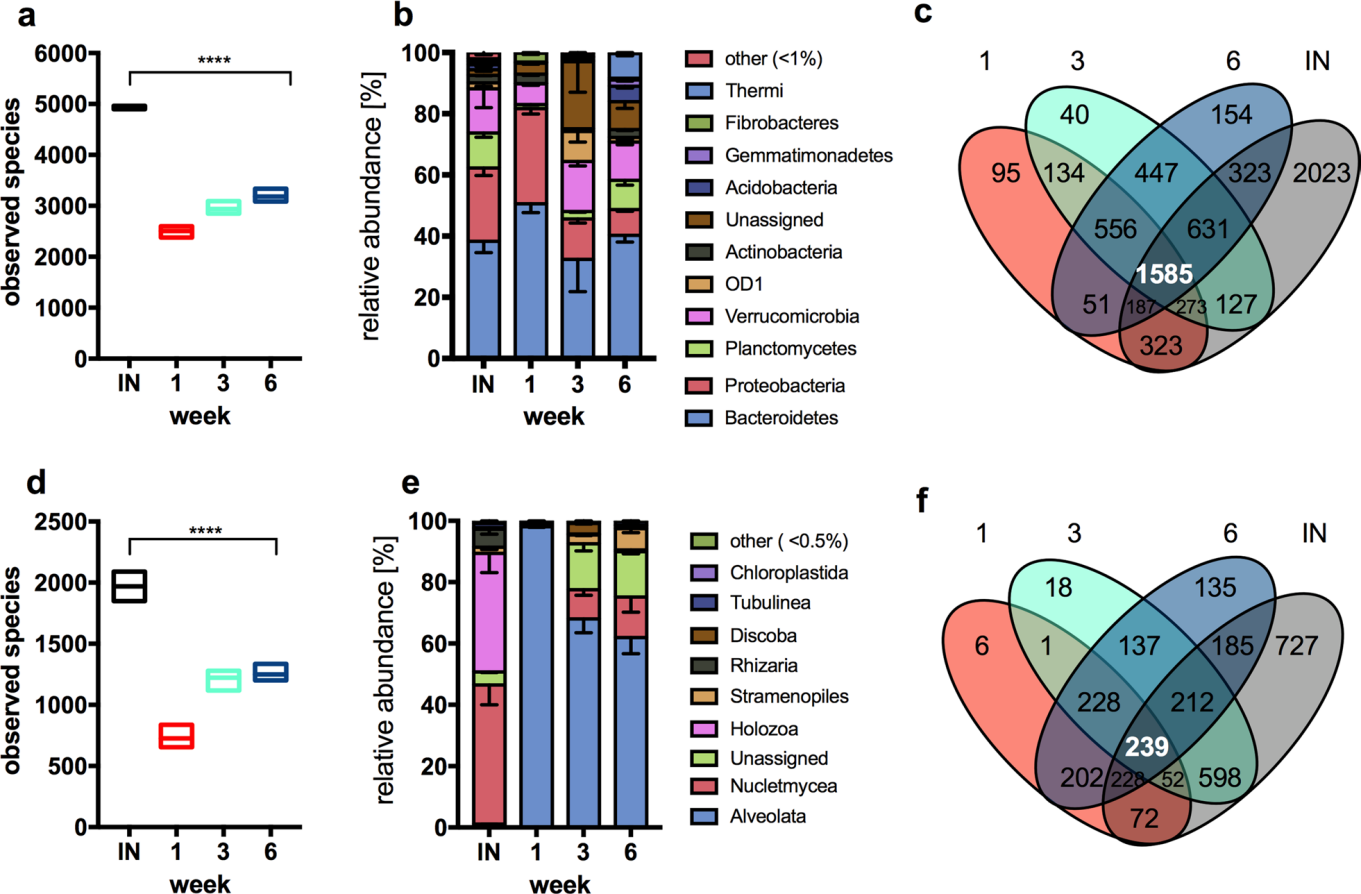
Taxonomic distribution of bacterial and eukaryotic community obtained by rRNA amplicon sequencing. **a** and **d** Number of observed species in wheat straw compost inoculum (IN) and compost-derived samples collected at week 1, 3 and 6. Floating bars represent minimum, mean and maximum number of detected OTUs. Ordinary one-way ANOVA test was performed to show significant differences in bacterial [**a**, F(3, 7) = 174, *p* < 0.0001] and eukaryotic [**d,** F(3, 7) = 57.11, *p* < 0.0001] richness between samples. **b** and **e** Relative abundance of the bacterial phyla and highest eukaryotic rank in inoculum (IN) and samples collected at week 1, 3 and 6. Error bars represent s.e.m, *n* = 3. **c** and **f** Venn diagrams represent number of shared and unique bacterial (**c**) and eukaryotic (**f**) OTUs between wheat straw compost inoculum (IN) and weekly samples (week 1, 3 and 6)

The highest number of bacterial (Fig. 2a) and eukaryotic OTUs (Fig. 2d) was detected in the compost inoculum, with an average of 4930 and 1970 OTUs, respectively, across two biological replicates. This was reflected by the inoculum showing the highest microbial diversity and richness based on alpha diversity estimation and rarefaction analysis (Additional file 2: Fig. S2) and a higher number of identified unique OTUs compared to the liquid culture samples (Fig. 2c, f).

The eukaryotic community within the compost inoculum was dominated by Metazoa (36.7%) with the major phyla being *Annelida* (annelid worms) and *Nematoda* (roundworms) accounting for 52 and 36% reads of this division. Fungi were also observed at high abundance within the inoculum, accounting for 35.5% of reads, 81% of which were identified as belonging to the *Ascomycota* lineage and 9% the *Basidiomycota* (Fig. 2e). Significant changes, however, in the eukaryotic community were observed under liquid growth conditions with both a decrease of identifiable OTUs (*n* = 3, Additional file 1: Table S1) detected in the first week post-inoculation, and a change in lineage composition (Fig. 2e). Metazoan and fungal assigned OTUs decreased to a level of < 1% 1 week after inoculation, with an increase in protozoa, of which 98% of OTUs were classified as belonging to the ciliate genus *Telotrochidium*.

Similarly, a decrease in diversity and richness of the prokaryotic community was observed once the compost inoculum was transferred into a liquid environment, reducing from ca. 5000 predicted OTUs in the inoculum (*n* = 2) to an average of ca. 2800 OTUs in the liquid cultures (*n* = 9, Additional file 1: Table S1). The compositional change in the prokaryotic community, after inoculation, was less affected than the eukaryotic community, and throughout the experiment, the majority of the 6949 bacterial OTUs detected from the lignocellulose degrading community were assigned to three major lineages: *Bacteroidetes* (40%), *Proteobacteria* (18%) and *Verrucomicrobia* (12.3%). To a lesser extent, *Planctomycetes* (5.7%) and *Thermi* (2.5%) were also present within the community. Although *Thermi* decreased in relative abundance during the first week post-inoculation, they went on to recover in number by the sixth week (Fig. 2b). The three most abundant bacterial genera in week 1, 3 and 6 were, respectively, *Asticcacaulis* (6.2%), *Leadbetterella* (5.1%) and *Truepera* (2.5%).

### General overview of the metatranscriptome and metasecretome

To investigate functional activity of the wheat straw enriched community, we sequenced the metatranscriptome (Additional file 1: Table S2, Additional file 2: Fig. S3) and analysed the metasecretome using the resulting metatranscriptomics data as a reference database for the spectra searches (Additional file 1: Table S3). We identified 300 proteins by LC–MS/MS analysis across all the time points within the culture supernatant (week 1, 3 and 6). In addition to this and to ensure that our analysis included proteins that had bound tightly to the insoluble components of the wheat straw cultures, we utilized an EZ-Link Sulfo-NHS-SS-Biotin label [23]. EZ-Link Sulfo-NHS-SS-Biotin is a membrane-impermeable probe, which crosslinks spontaneously to exposed primary amines, enabling the labelling of extracellular proteins and subsequent purification by affinity chromatography after stringent washing of the biomass samples. From this analysis, 1127 distinct proteins were identified, 990 of which were unique to the biotin-labelled samples. Furthermore, hierarchical clustering analysis differentiated metasecretomes of supernatant and biotin-labelled fractions into two separate groups and confirmed the reproducibility of the methodology (Additional file 2: Fig. S4).

To compare the functionality of the metasecretome to metatranscriptomics data, we analysed the 10,000 most abundant transcripts that were selected based on their normalized Expressed Sequence Tag (EST) counts. The relative abundance of Clusters of Orthologous Groups (COGs) in metatranscriptome and metasecretome data showed similar functional distributions for most of the groups with the enrichment of genes in the metatranscriptome involved in replication, recombination and repair (ratio = 4.5) and defense mechanisms (ratio = 3.9, Additional file 2: Fig. S5).

BLASTp searches, however, demonstrated that the proteomic samples from the biotin-labelled fractions contained a number of transporters and membrane-associated proteins (Additional file 3). TonB-dependent (TBDT), adenosine triphosphate (ATP)-binding cassette (ABC) and tripartite ATP-independent periplasmic (TRAP) transporters accounted for 165 annotated proteins (20%). The variety of transporters and membrane proteins (such as OmpA/MotB-containing proteins) expressed by the microbial community indicates a variety of nutritional strategies, which will benefit the microorganism when sugar and amino acids monomers/oligomers are available for uptake. Transporters and membrane-associated proteins were reported as ubiquitous in other metasecretomes of complex communities [18, 24]. Other abundant functional groups were proteins required for intracellular trafficking, secretion, and vesicular transport (7.1%) and cell wall/membrane envelope biogenesis and motility (11.6%).

### Phylogenetic origin of putative proteins

The phylogenetic origin of all the putative proteins (as assessed by sequence similarity) showed that two major phylogenetic groups: Bacteria (80.2%, s.d. = 5.1) and Eukaryota (10.6%, s.d. = 5.1) contributed to the metasecretomes. Consistent with the community analysis described previously, the proteins assigned to Eukaryota originated mainly from the Alveolata group and orders *Hymenostomatida* (including *Tetrahymena*) and *Peniculida* (including *Paramecium*). Of the 65 proteins identified as belonging to *Tetrahymena* and *Paramecium*, only six were present in the supernatant, and all but two were preferentially present within the biotin-labelled fraction. These proteins were not predicted to have lignocellulose-degrading capabilities, and instead 30.4% were predicted to have protease activities and 15.3% were assigned as hypothetical.

The majority of prokaryotic proteins identified were affiliated with phyla *Proteobacteria* and *Bacteroidetes*. These accounted for 65.7% (s.d. = 12.9) and 22.3% (s.d. = 9.1) of the proteins detected in the metasecretome, respectively. Proteins derived from orders *Xanthomonadales* (29.8%) and *Rhizobiales* (20.7%) were the most abundant in the *Proteobacteria* phylum. Several genera such as *Leadbetterella* (*Bacteroidetes*) and α-proteobacteria *Devosia*, *Mesorhizobium* and *Rhizobium* contributed 30% of putative transporters identified in the metasecretome suggesting an ability to transport nutrients more rapidly than other microorganisms in the cultures. Overall, the metasecretome proteins were predicted to be produced by > 250 genera with the majority being of bacterial origin indicating a high microbial diversity that accounts for the functionality of the community (Additional file 3).

### Analysis of community lignocellulose degrading capability in early, mid and late stages of the wheat straw cultures

**Fig. 3:**
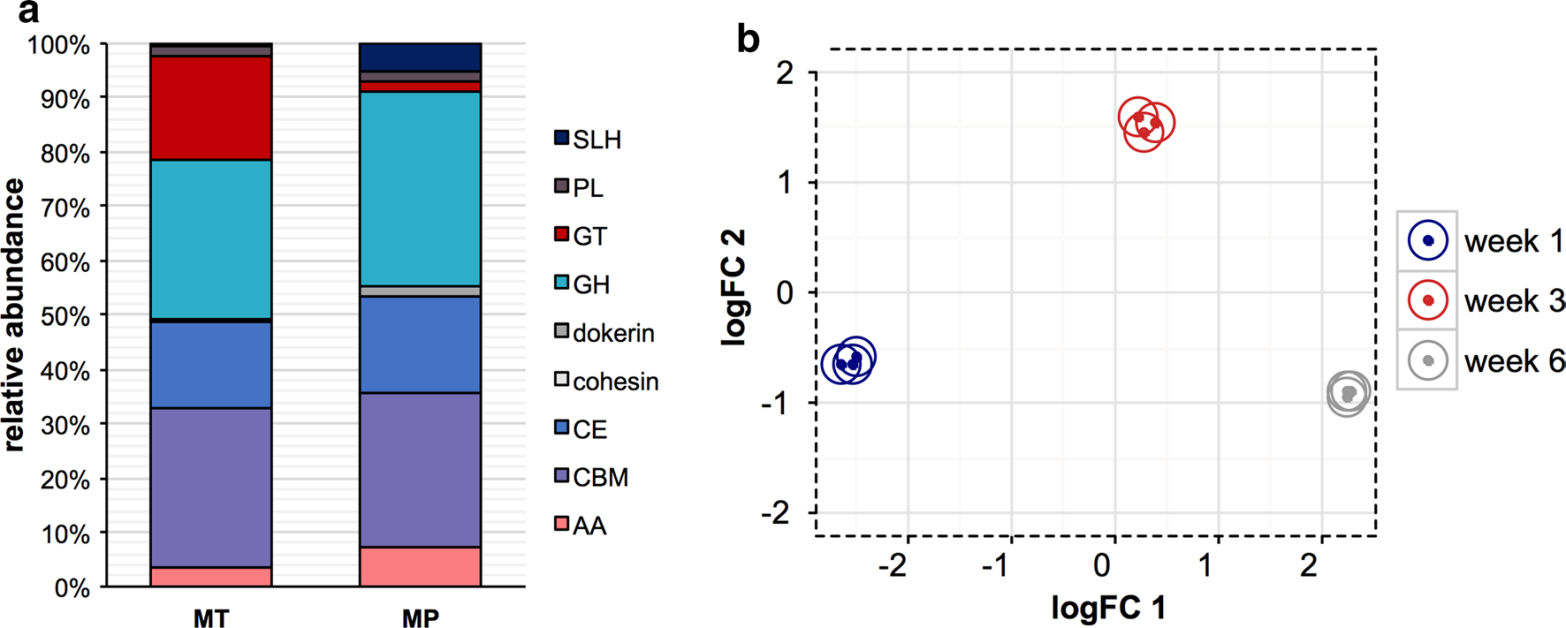
Overview of proteins and transcripts assigned to CAZymes. **a** Relative abundance of CAZy families identified in metatranscriptome (MT) and metasecretome (MP) of wheat straw compost-derived communities. **b** Multi-dimensional scaling (MDS) plot of the transcripts encoding proteins identified in metasecretome of wheat straw compost-inoculated cultures. Distances correspond to leading log-fold-changes between each pair of RNA samples. The leading log-fold-change is the average (root-mean-square) of the largest absolute log-fold changes between each pair of samples

Both the wheat straw loss of mass and qualitative analysis of its cell wall composition showed that rapid carbohydrate degradation was occurring within the liquid cultures. To identify the carbohydrate-active enzymes in the metasecretome, we performed a similarity search of the identified proteins from the microbial community against the entire non-redundant sequences of the CAZy database using the dbCAN server (Fig. 3a, Table 1). Using this analysis, we demonstrated the presence of 52 proteins assigned as putative CAZymes within the wheat straw degrading community.

**Table 1:** Carbohydrate-active enzymes identified in the metasecretome of a wheat straw degrading compost-derived community

Protein ID	Predicted domain^a^	Predicted CAZy class	Description	Hit accession	E-value	Seq ID (%)	Coverage (%)	Taxonomic affiliation	Taxonomic kingdom
Proteins unique to biotin-labelled fraction									
c134278_g1_i1_1	CBM2-CBM60-GH10	GH	Beta-1,4-xylanase	BAD88441	0	86	73	*Pseudomonas*	Bacteria
c155626_g1_i1_1	CE10	CE	Esterase	WP_051322721	4E-152	77	100	*Luteimonas*	Bacteria
c155626_g1_i1_4	GH51	GH	Alpha-N-arabinofuranosidase	WP_024870432	0	80	100	*Pseudoxanthomonas*	Bacteria
c180274_g1_i1_4	GH5	GH	Endoglucanase	AIF91557	0	61	83	*Alteromonadaceae*	Bacteria
c180864_g1_i1_2	CBM2-CBM22-GH10	GH	Endo-1,4-beta-xylanase	WP_007643754	0	71	99	*Cellvibrio*	Bacteria
c189421_g1_i1_1	GH6	GH	Cellobiohydrolase	WP_013117127	0	95	92	*Cellulomonas*	Bacteria
c190646_g3_i1_5	CBM2-CBM10-GH6	GH	Cellobiohydrolase	ACE85978	0	67	100	*Cellvibrio*	Bacteria
c190817_g1_i1_7	CBM44	CBM	Hypothetical protein	WP_052773020	0	65	90	*Luteimonas*	Bacteria
c192149_g1_i1_4	CE10	CE	Peptidase S9	WP_027072364	0	79	91	*Luteimonas*	Bacteria
c204578_g3_i1_1	CBM2-CBM22	CBM	Endo-1,4-beta-xylanase	WP_007643754	0	84	98	*Cellvibrio*	Bacteria
c207114_g1_i1_5	GH38	GH	Hypothetical protein	WP_051602586	5E-111	35	85	*Hyphomonas*	Bacteria
c207852_g16_i3_8	CE10	CE	Hypothetical protein	WP_027072315	0	84	95	*Luteimonas*	Bacteria
c208411_g1_i3_1	CBM61	CBM	Subtilisin-like proprotein	WP_014780843	1E-18	37	25	*Aequorivita*	Bacteria
c208914_g1_i1_3	CE10	CE	Unknown	CDW81391	3E-42	64	98	*Stylonychia*	Eukaryota
c209736_g2_i3_67	GT4	GT	Hypothetical protein	WP_005674657	1E-115	49	99	*Lautropia*	Bacteria
c209807_g2_i1_6	SLH	SLH	S-layer protein	WP_013176560	2E-166	37	100	*Truepera*	Bacteria
c211555_g1_i1_2	GH11-CBM60	GH	1,4-beta-xylanase	WP_049629015	0	81	100	*Cellvibrio*	Bacteria
c27111_g1_i1_1	CBM2-CBM2	CBM	Hypothetical protein	ESQ13017	7E-111	39	99	Uncultured desulfofustis	Bacteria
c344648_g1_i1_2	CBM50	CBM	Peptidoglycan-binding protein	WP_027071529	0	88	99	*Luteimonas*	Bacteria
c345827_g1_i1_2	CE15	CE	Hypothetical protein	WP_012238067	0	68	83	*Sorangium*	Bacteria
c528853_g1_i1_2	CBM44	CBM	CARDB domain-containing protein	AEV33755	4E-52	34	51	*Owenweeksia*	Bacteria
c711977_g1_i1_3	SLH	SLH	Hypothetical protein	WP_009455020	1E-14	31	29	*Fischerella*	Bacteria
c724886_g1_i1_1	CBM2-GH5	GH	Endo-1,4-beta-D-glucanase	ACY24859	0	81	100	Uncultured microorganism	
c80983_g1_i1_2	CBM2-CBM60-CE1	CE	Hypothetical protein	WP_051234546	0	74	99	*Marinimicrobium*	Bacteria
c34457_g1_i1_2	AA2	AA	Catalase/peroxidase HPI	WP_036397899.1	2E-78	1	0.9	*Mycobacterium*	Bacteria
c210210_g2_i1_1	GH109	GH	gfo/Idh/MocA family oxidoreductase	WP_006979342.1	9E-151	0.99	0.52	*Chthoniobacter*	Bacteria
c350217_g1_i1_3	AA6	AA	NADPH-dependent FMN reductase	WP_027072629.1	3E-100	1	0.8	*Chthoniobacter*	Bacteria
c180629_g1_i1_1	GH25	GH	glycosyl hydrolase family 25 protein	XP_001008527.1	6E-64	0.98	0.5	*Tetrahymena*	Eukaryota
c190646_g2_i1_3	CBM2	CBM	Cellobiohydrolase	WP_007642349.1	0	1	0.74	*Cellvibrio*	Bacteria
c208441_g1_i1_2	GH3	GH	1,4-beta-D-glucan glucohydrolase	WP_027070958.1	0	0.97	0.82	*Luteimonas*	Bacteria
c151435_g1_i1_2	GH5	GH	Endoglucanase	WP_084618390.1	0	1	0.74	*Cellvibrio*	Bacteria
c199479_g2_i1_4	CBM2	CBM	DUF1592 domain-containing protein	WP_007644728.1	0	1	0.78	*Cellvibrio*	Bacteria
c207123_g9_i1_7	CE1	CE	S9 family peptidase	WP_043740359.1	0	0.95	0.87	*Luteimonas*	Bacteria
c349698_g1_i1_1	GH9	GH	Glycoside hydrolase	WP_041523229.1	0	1	0.7	*Gilvimarinus*	Bacteria
c203693_g1_i1_7	CBM4	CBM	Cellulose 1,4-beta-cellobiosidase	WP_085113009.1	7E-19	0.39	0.26	*Thermoanaerobacterium*	Bacteria
c225675_g1_i1_1	CBM44	CBM	T9SS C-terminal target domain-containing protein	WP_041627631	2E-52	32	59	*Owenweeksia*	Bacteria
Proteins unique to supernatant fraction									
c159637_g1_i1_5	DOCKERIN	Dockerin	n.d						
c205510_g2_i1_1	GH74	GH	T9SS C-terminal target domain-containing protein	WP_027376910	7E-41	55	100	*Chryseobacterium*	Bacteria
c38599_g1_i1_1	CBM37	CBM	Hypothetical protein	WP_035755077.1	2E-142	1	0.55	*Flavobacterium*	Bacteria
c203621_g1_i1_2	CBM6	CBM	T9SS C-terminal target domain-containing protein	WP_040481137.1	1E-86	0.32	0.5	*Mariniradius*	Bacteria
c185673_g1_i1_1	AA6	AA	NADPH-dependent oxidoreductase	WP_046482965.1	9E-109	0.99	0.81	*Pseudomonas*	Bacteria
c208949_g1_i1_1	PL9-PL9	PL	Nitrous oxidase accessory protein	WP_014202187	3E-19	25	30	*Owenweeksia*	Bacteria
Proteins common between biotin-labelled and supernatant fractions									
c155243_g1_i1_4	GH38	GH	TonB-dependent receptor	WP_052633156	0	79	99	*Pseudoxanthomonas*	Bacteria
c186013_g1_i1_1	GH6	GH	Cellobiohydrolase	WP_027328555	0	77	84	*Marinimicrobium*	Bacteria
c194919_g2_i1_5	CBM2	CBM	Carbohydrate-binding protein	WP_007644728	0	77	99	*Cellvibrio*	Bacteria
c208473_g1_i1_1	CE8-CBM37	CBM	T9SS C-terminal target domain-containing protein	WP_028522307	4E-74	55	49	*Runella*	Bacteria
c194919_g2_i1_4	CBM2	CBM	YceI family protein	WP_087469052.1	0	0.51	1	*Cellvibrio*	Bacteria
c209441_g2_i1_1	AA7	AA	FAD-binding protein	WP_053231510.1	6E-164	0.93	0.51	*Sandaracinus*	Bacteria
c711379_g1_i1_1	GH74	GH	T9SS C-terminal target domain-containing protein	WP_084016764.1	0	1	0.94	*Moheibacter*	Bacteria
c531189_g1_i1_2	CE10	CE	Hypothetical protein	PCJ83084.1	5E-116	1	0.39	*Flavobacteriales*	Bacteria
c205510_g1_i1_1	GH74	GH	T9SS C-terminal target domain-containing protein	WP_084016764.1	0	1	0.83	*Moheibacter*	Bacteria
c63252_g1_i1_3	GH48	GH	Exoglucanase	WP_013118318.1	0	0.93	0.93	*Cellulomonas*	Bacteria
c349698_g1_i1_5	CE1	CE	Feruloyl esterase	WP_072812830.1	2E-67	0.88	0.43	*Fibrobacter*	Bacteria
c540340_g1_i1_1	CBM44	CBM	T9SS C-terminal target domain-containing protein	WP_070137948.1	1E-42	0.56	0.6	*Crocinitomix*	Bacteria
c190535_g1_i1_1	SLH	SLH	Hypothetical protein	OGI23348.1	2E-140	1	0.39	n.d	Bacteria
c600504_g1_i1_1	CE8	CE	n.d						

^a^Domain structure for each protein was predicted based on CAZy database searches

Degradation of lignocellulosic biomass, such as wheat straw, is governed by the combined action of modular glycosyl hydrolases, lytic polysaccharide monooxygenases (LPMOs) and lignin-modifying enzymes. Proteins containing glycosyl hydrolase domains, as annotated by the dbCAN server, were the most abundant accounting for 26 of the 52 CAZymes identified. This was followed by 16 carbohydrate esterases, two glycosyltransferases, one pectin lyase and seven proteins identified as belonging to the auxiliary activities grouping. Carbohydrate-binding modules (CBMs) were identified on twelve of the predicted CAZyme proteins; seven CBMs were associated with glycoside hydrolase assigned proteins and five CBMs were appended with carbohydrate esterase domains. An additional 24 proteins contained CBMs but they were linked with no identifiable CAZyme domain. A summary of these proteins and their closest BLAST hits is shown in Table 1. All putative CAZymes were of bacterial origin, apart from a carbohydrate esterase that showed similarity to a protein from the protozoan ciliate *Stylonychia,* and a lysozyme (GH25) affiliated with *Tetrahymena* family, of protozoan ciliates. Proteobacteria of the genera *Cellvibrio* and *Luteimonas* made the largest contribution to detected CAZymes in the metasecretome by producing 30% of identified putative CAZymes.

The molar abundance of the CAZyme-assigned proteins in both the biotin-labelled fraction and the supernatant accounted for 1.79% of the total metasecretome. The importance of analysing the biotin-labelled fraction was demonstrated by the greater diversity of CAZyme- assigned proteins observed compared to the supernatant fraction alone. Just eighteen of the 52 proteins with CAZyme domains were identified in the supernatant, whilst 48 were identified in the biotin-labelled fraction. There was, however, no significant difference in molar abundance of CAZymes detected between these samples, in part due to the abundance of a predicted pectinase that was found in supernatant fractions at a molar abundance of 0.46% in comparison to 0.05% in the biotin-labelled fractions.

**Fig. 4:**
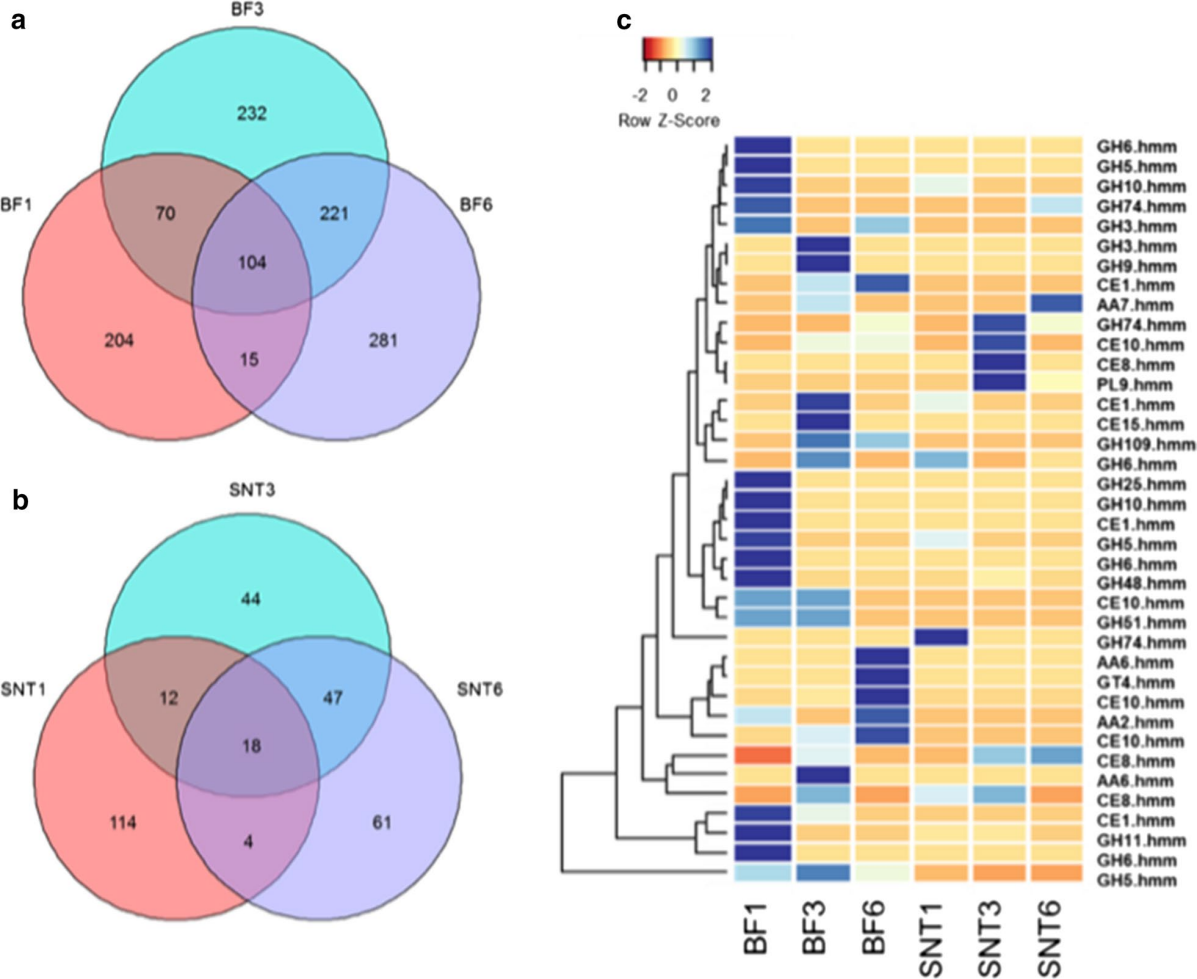
Overview on metasecretome from wheat straw degrading compost-derived community. **a** and **b** Venn diagrams on unique and shared proteins in biotin-labelled (BF) and supernatant (SNT) fraction from week 1, 3 and 6. **c** Distribution of carbohydrate-active enzymes across the 6-week course in biotin-labelled (BF) and supernatant (SNT) fractions, rows are coloured by the *z*-score of the molar percentage of each detectable protein, and the attached dendrogram displays Bray–Curtis clustering

We then analysed the production of lignocellulose-degrading enzymes by the wheat straw degrading microbial community over time (Fig. 4). In the early stage of wheat straw degradation, the community produced xylanases and cellulases from families GH 5, 6, 10, 11. These were predominantly identified within the biotin-labelled metasecretome fractions and represented a total of 1.32% of the molar abundance of identified proteins in the first week. The relative abundance of these putative xylan and cellulose degrading enzymes receded throughout the time course, falling to 0.51% by the third week and 0.17% by the sixth. A putative GH5 cellobiohydrolase from the Proteobacteria genus *Cellvibrio* was the most abundant glycoside hydrolase in the biotin-labelled samples, and the only hypothetical cellulase to persist in the cultures throughout the 6-week culture period without significant reduction. Interestingly, this protein contains a long serine repeat at the N-terminus—a characteristic that was found in multiple proteins within the secretome, all of which were predicted to have lignocellulolytic or hypothetical functions.

In contrast to the first week of incubation, when proteins with similarities to cellulose and xylan degrading enzymes were dominant, the two most abundant CAZymes in the third week were identified as belonging to the CE8 family, a family whose known members are exclusively pectin methylesterases. This apparent upregulation of proteins relating to pectin degradation in the third week was also demonstrated by the appearance of a polysaccharide lyase (PL9), which has been characterized as containing activities including pectate degradation. By the third week of incubation, the abundance of chitinases, and enzymes relating to peptidoglycan degradation had also significantly increased, suggesting a turnover of microbial biomass within the community.

Similarly, the metatranscriptome database was searched for CAZy-encoding genes. Overall, 4632 ORFs (1.37% of the total predicted ORFs in the metatranscriptome) were assigned as putative carbohydrate-active enzymes belonging to 218 different CAZy families. The transcripts containing carbohydrate-binding modules (CBM) family 44 were the most abundant (7.8%) CAZymes in the metatranscriptome. Family CBM44 is poorly characterized but is predicted to participate in binding cellulose/xyloglucan. The second most abundant group of CAZymes in the metatranscriptome was CBM50, which are attached to many GHs that cleave either chitin or peptidoglycan. In contrast, the third most abundant group of CAZyme transcripts encoded glycosyltransferases (GT) from family 2 (6.8%). Members of this GT family show activity towards the synthesis of various oligo- and polysaccharides including cellulose, chitin, and peptidoglycan. Amongst the most abundant glycosyl hydrolases were α-N-acetylgalactosaminidase from GH109 (4.1%) and lysozymes from GH18 and GH23, which suggests significant competition within the community in agreement with the previously described meta-proteome.

## Discussion

Lignocellulose degrading communities in environments like soil and compost are known to contain organisms that represent all domains of life and are involved in nutrient recycling including heterotrophic carbon consumption and turnover of fixed carbon during degradation of plant biomass [7, 19]. In this study, we used compost to inoculate shake flasks containing wheat straw as the sole carbon source. A significant reduction of the wheat straw biomass demonstrated that the microbial community had successfully colonized and utilized wheat straw as a carbon source. An increase in pH was observed over the course of the incubations. The alkalization during lignocellulose degradation has been reported previously with the degradation of carboxylic acids and phenols causing an increase in pH from neutral range to alkaline. It has also been reported that although lignocellulose mineralization occurs preferably in neutral or slightly acid environments, the lignin solubilization and lignin by-products release are greatest when the pH is alkaline [25, 26]. In addition, studies have shown that multiple carbohydrate-active enzymes express alkaliphilic behaviour and are most active in higher pH ranges [27, 28]. Increase in external pH in our batch cultures might be also attributed to protein degradation as excess ammonia generated during amino acid catabolism may be secreted into the medium. Furthermore, the bacterial community in liquid cultures with a limited and hard to access carbon source are likely to have a decreased respiration rate that would prevent acidogenesis [29].

To identify key members of the lignocellulose degrading community and monitor the changes in the community composition at sampled time points, amplicon sequencing of 16S and 18S SSU rRNA was performed. Targeted-amplicon sequencing of these regions has been widely used to predict relative abundance of prokaryotic and eukaryotic microorganisms [3, 5, 30, 31]. The number of OTUs constructed during this study is significantly higher than reported elsewhere [30, 32], and the depth of sequencing approach increased the sensitivity of detecting OTUs giving a deeper insight into the complexity of the microbial community.

The most striking changes to the community structure occurred during the transfer of the solid-state compost inoculum to liquid cultures. The eukaryotic community in the compost inoculum resembled forest soil communities [33] with a small contribution from the ‘protists’ group. This community structure underwent a dramatic transformation once transferred to liquid medium. Fungi, which are one of the major contributors to lignocellulose degradation in the terrestrial environment, were seen to decrease to less than 1% of the total OTUs [34, 35], whilst the eukaryotic community in the liquid cultures was instead dominated by protists. These protists were assigned to the genus *Telotrochidium*, a peritrich ciliate which is known to thrive in liquid environments [36]. In contrast, many of the fungal OTUs which dominated the inoculum were from the phylum *Ascomycota* and as such are expected to be xerophilic [37], and hence transfer to the liquid medium may have impaired their growth. In addition to changing the community composition, transfer from the solid state to a liquid culture also reduced the number of distinct OTUs detectable within both the eukaryotic and prokaryotic communities as a result of the enrichment process. The three most abundant bacterial genera were *Asticcacaulis*, *Leadbetterella* and *Truepera*. The genus *Asticcacaulis* has been found to be highly abundant in lignocellulolytic consortia from decaying wood, canal sediment, soil and forest litter and composted sugarcane bagasse [38, 39]*. Asticcacaulis* produce lignocellulose-modifying enzymes such as glucosidases, galactosidases and xylosidases and actively degrade plant materials [38]. Similarly, *Bacteroidetes* of genus *Leadbetterella* are capable of degrading starch and a variety of other saccharides and have previously been isolated from cotton waste compost and samples associated with lignocellulose degradation in leaf-cutter ant refuse dumps [40, 41]. Finally, a higher abundance of *Thermi* in week 6 was possibly due to the depletion of easily available carbon sources and the increasing pH. The genus *Truepera* belonging to the *Deinococcus*-*Thermus* phylum is less often associated with plant cell wall degradation but has recently been reported in compost enrichment samples [6, 42]. *Truepera* isolates are known to resist harsh growth conditions including high pH and temperature and exposure to toxic compounds [43], which could drive their proliferation in the wheat straw liquid cultures in later stages of the experiment.

Though the protists dominated the eukaryotic community within the cultures, the extent of which they are directly involved in lignocellulose degradation remains unclear. Though some protists and their bacterial endo-symbionts have been reported as active producers of lignocellulolytic enzymes in the termite gut [44] and cow rumen [45], with no known lignocellulose degrading proteins from protist detected in the metasecretomes their role in the wheat straw liquid cultures may be predatory in line with previously reported results [46, 47].

Much of the functionality of lignocellulose degradation appeared to be driven by the prokaryotic community. The dominance of *Bacteroidetes* and *Proteobacteria* in the wheat straw cultures is similar to previous studies [5], and although the representation of *Proteobacteria* decreased during the time course, members of this phylum, specifically from orders *Pseudomonadales*, *Rhizobiales* and *Xanthomonadales* contributed to the majority of the secreted proteins. Here, the genera *Cellvibrio* and *Luteimonas* appeared to contribute significantly to lignocellulose degradation by providing array of CAZymes, in line with previous studies [15]. These genera, however, appeared to be present in low relative abundance based on the 16S evaluation, whilst genera that produced the most membrane transporters, such as *Leadbetterella* (Bacteroidetes) and α-proteobacteria *Devosia*, *Mesorhizobium* and *Rhizobium*, accounted for the majority of the 16S sequencing reads.

The release of carbon from the wheat straw over time appeared to be sequential. In the first week, consistent with the observation that both the xylanase and cellulose activities peak within this time point, the majority of cellulases and xylanases related proteins are found within the metasecretome. Subsequently, a rise in the number of proteins related to chitin and pectin degradation occurred 3 weeks post-inoculation, perhaps signifying a change in nutrient acquisition strategy, as the easily accessible plant cell wall polysaccharides are removed. Wheat straw contains c.a. 5% of pectin, which is partially acetylated and has a low level of methoxylation but a moderate content of galacturonic acid (25%). Alkaline pre-treatment studies on wheat straw showed a higher removal rate of hemicellulose than pectin fraction suggesting that this polysaccharide is more recalcitrant to degradation [48]. Others also showed that reduction of de-methyl-esterified homogalacturonan pectin in plants increases the efficiency of enzymatic saccharification, reducing the need of biomass pre-treatment [49–51]. The observation that pectin-related enzymes, such as those belonging to the CE8 family and pectin lyase class, are present 3 weeks post inoculation is intriguing, however, not unprecedented as pectinolytic enzymes have been reported as being prevalent in composting microbial communities [7, 52].

Other interesting targets for further characterization are 24 proteins with predicted carbohydrate-binding modules and no identifiable CAZyme domain attached, raising the possibility for the discovery of new enzymatic domains.

Further targets for the discovery of new lignocellulose degrading enzymes were identifiable using a biotin affinity-labelling that targets proteins bound to wheat straw via carbohydrate-binding modules. We previously described the advantage of this affinity tagging approach to identify lignocellulose active proteins bound tightly to their substrates which render them unobservable using traditional extraction techniques [23]. By sequencing the biotin-labelled fraction, we were able to increase the number of carbohydrate-active enzymes observed in our analysis by over 250%.

## Conclusion

In summary, the work presented here offers an insight into the microbial digestion of wheat straw, and dynamic microbial communities that govern the process. Though much of the full functionality of the microbial community remains unclear, due to the complex nature of the samples and the difficulties in assigning divergent sequences functions, broad patterns of lignocellulolytic enzyme production have been described throughout a 6-week time course, along with the identification of proteins which could convey novel functions against lignocellulose.

## Materials and methods

### Compost liquid cultures

Wheat straw compost that had been enriched by periodic addition of straw over a period of 3 months was used as an inoculum for the cultures. The compost was mixed thoroughly and homogenized using an electric blender. Flasks (2 litre) containing 700 ml of mineral medium (per litre: KCl—0.52 g, KH_2_PO_4_—0.815 g, K_2_HPO_4_—1.045 g, MgSO_4_—1.35 g, NaNO_3_—1.75 g, 1000 × Hutner’s trace elements—1 ml, pH 6.2) and 5% (w/v) wheat straw as a sole carbon source were inoculated with 1% (w/v) of the compost material. Cultures (three independent biological replicates were used) were incubated at 30 °C at 150 rpm for 8 weeks. Weekly aliquots (50 ml) were harvested to collect wheat straw biomass and culture supernatant by centrifugation at 4000×*g* at 4 °C for 10 min. The latter fractions were processed separately for nucleic acids and protein extraction.

### Nucleic acid extraction and purification

Total nucleic acids (DNA and RNA) extraction from freshly collected aliquots of the wheat straw cultures were performed using an adapted protocol by Griffiths et al. [53]. Briefly, 0.5 g of wheat straw material from the liquid cultures was lysed by bead beating (Qiagen TissueLyser II, 2.5 min at speed 28/s) in a presence of 0.5 ml CTAB solution (10% CTAB in 0.7 M NaCl, 240 mM potassium phosphate buffer pH 8.0) and 0.5 ml of phenol:chloroform:isoamyl alcohol (25:24:1, pH 8.0). Subsequently, tubes were centrifuged at 16,000×*g* for 5 min at 4 °C, the aqueous phase was collected and extracted with 1 volume of chloroform: isoamyl alcohol (24: 1). The nucleic acid was precipitated by adding 2 volumes of 1.6 M NaCl/20% PEG8000 buffer and samples were incubated overnight at 4 °C. The pellet was washed twice with ice-cold 70% ethanol and once dried was resuspended in RNase/DNase free water. Samples were stored at − 80 °C.

### Processing DNA samples for community profiling and analysis

Prokaryotic primers S-D-Bact-0564-a-S15 (AYTGGGYDTAAAGNG) and S-D-Bact-0785-b-A-18 (TACNVGGGTATCTAATCC) [54] and eukaryotic primers F1422 (ATAACAGGTCTGTGATGC) and R1631 (TACAAAGGGCAGGGACGTAAT) [55] were used to amplify the 16S and 18S SSU rRNA genes, respectively. The reactions were carried out in 50 µl volumes containing 200 µM of dNTPs, 0.5 µM of each primer, 0.02 U Phusion High-Fidelity DNA Polymerase (Finnzymes OY, Espoo, Finland) and 5× Phusion HF Buffer containing 1.5 mM MgCl_2_. The following PCR conditions were used: initial denaturation at 98 °C for 1 min, followed by 30 cycles consisting of denaturation (98 °C for 10 s), annealing (30 s) and extension (72 °C for 15 s) and a final extension step at 72 °C for 5 min. Annealing temperature for 16S primer pair was set at 42 °C and for 18S primer pair was set to 53 °C. The expected amplicon sizes for 16S and 18S rRNA gene were 207 and 180 bp, respectively. The quantity and quality of the purified PCR products were analysed by Agilent Tape Station using Agilent DNA 1000 kit. The sequencing was performed by the Biorenewables Development Centre (BDC, York) using the Ion Torrent platform. The individual sequence reads were filtered using the PGM software to remove low-quality reads and polyclonal reads. Sequences matching the PGM barcodes were trimmed and FastQ format files were produced for processing using QIIME 1.8.0. The split_library.py script was applied to remove primers, exclude poor quality reads (Q < 25) and sequences outside the defined read length. The chimeric sequenced were removed using usearch61. The remaining non-chimeric sequences were clustered by pick_open_reference_otus.py into OTUs (Operational Taxonomic Units) at 97% similarity using UCLUST as a clustering method. The bacterial OTUs were taxonomically annotated using Greengenes (gg_13_8, March 2015) database; the eukaryotic OTUs were assigned to taxonomy using Silva_119 database. Biom-formatted OTU tables were created and filtered to exclude OTUs containing fewer than ten sequences. Alpha diversity was evaluated by rarefaction curves to the maximum sequence depth obtained per sample and additionally by calculating indices including: Chao1 richness, Shannon and Simpson diversity and number of OTUs (observed_species) using relevant QIIME scripts. Raw reads in FastQ format were submitted to the European Nucleotide Archive (ENA) and are available under accession number PRJEB21053.

### Processing RNA samples for metatranscriptomics, sequencing and analysis

RNA extracted from the samples was treated with RTS DNase (MoBio), followed by elimination of small RNAs and purification (Zymo Research). The quality and quantity of RNA samples was assessed using an Agilent Tape Station with Agilent RNA screen tape. A 2.5 µg sample of total RNA was used for mRNA enrichment (Epicentre Epidemiology). Ribosomal RNA-depleted samples were purified (Zymo Research) and their profile assessed by Agilent Bioanalyser mRNA analysis. Samples were sequenced at the Earlham Institute (previously TGAC, Norwich, UK) using Illumina HiSeq 2500 technology. Nine 2× 100 bp Illumina TrueSeq RNA libraries were generated (~ 327 million reads combined) with an average insert size of 425 bp (Additional file 1: Table S2). The sequenced libraries were searched against Silva_115 [56] database to identify ribosomal RNA genes using Bowtie2 software [57]. On average 36.46 ± 17.40% of the cDNA sequences were identified as ribosomal RNA ranging from 3.44 to 54.85%. As reported in other metatranscriptomic studies [58], the depletion of rRNA from the total RNA samples was not sufficient, especially in early time points, possibly due to unsuccessful removal of protozoan rRNA. Those reads as well as orphans and poor quality sequences were removed with the ngsShoRT software and pooled prior assembly with de novo Trinity package [59]. The total number of reads used for assembly was > 8.8 million which resulted in 998,793 contigs with an average length of 400 bp. 338,157 open reading frames (ORFs) were predicted using EMBOSS ORF finder. The abundance of each transcript from every sequenced library was defined as EST count which was subsequently normalized to count per million. The mapping of original individual libraries to the Trinity transcriptomic assembly was done with the BWA software to estimate raw counts for individual contig in each library. The wheat straw metatranscriptome has been deposited to the ENA and is available under accession number PRJEB12382.

### Processing protein samples for metasecretome analysis

Fractionated metasecretome samples of the wheat straw cultures were prepared and analysed as described previously [23]. Briefly, soluble extracellular proteins were obtained by precipitation with five volumes on ice-cold acetone of the culture supernatant, which was clarified and filtered sterilized (0.22 µm PES filter unit) prior extraction. The concentrated protein pellet was washed twice with 80% ice-cold acetone, air-dried and resuspended in 0.5 ×  PBS (68 mM NaCl, 1.34 mM KCl, 5 mM Na_2_HPO_4_, 0.88 mM KH_2_PO_4_, pH 8.0) buffer.

Cell wall bound and biomass adherent proteins were labelled using sulfo-NHS-SS-biotin and affinity purified. Two grams of washed (0.5 × PBS) wheat straw biomass was resuspended in 0.5 × PBS supplemented with 10 mM EZ-link-Sulfo-NHS-SS-biotin (Thermo Scientific). Following 1-h incubation at 4 °C on the rotator, the reaction was stopped by the addition of 50 mM Tris–HCl, pH 8.0. The residual biotin was removed by a biomass washing with ice-cold 0.5 × PBS (twice). For a total protein extraction, pre-warmed 2% SDS (60 °C) was added and samples were incubated for 1 h at room temperature. The mixture was centrifuged and proteins were precipitated as described above. Biotin-labelled protein pellets were solubilized in 1 × PBS (137 mM NaCl, 2.7 mM KCl, 10 mM Na_2_HPO_4_, 1.8 mM KH_2_PO_4_) containing 0.1% SDS, and passed through a 0.22-µm PES filter unit before being loaded onto pre-washed (0.1% SDS in 1 × PBS buffer) streptavidin columns (#GE17-5112-01, Thermo Scientific). Proteins were incubated on the column for 1 h at 4 °C to aid binding, before being washed with 0.1% SDS in 1 × PBS. Columns were incubated overnight at 4 °C with elution buffer of 50 mM DTT in 1 × PBS. Sequential elution of proteins from the streptavidin column was done by loading 4 times 1 ml 50 mM DTT in 1 × PBS, collecting the fraction and incubating the column for 1 h before next elution. Eluted fractions were freeze-dried, resuspended in 2 ml distilled water and desalted (Zeba, 7 K MWCO, Thermo Scientific). Supernatant and biotin-labelled protein samples were subjected to SDS-PAGE on 4–12% Bis–Tris gels, and protein bands were excised and cut into 1-mm pieces which were stored at − 80 °C prior to analysis.

LC–MS/MS analysis was performed and peptides were identified using the Mascot search engine against protein sequences from the metatranscriptomics database as described before [23].

### Wheat straw biomass analysis

Fourier transform infrared (FTIR) and nuclear magnetic resonance (NMR) analysis were carried out on ground freeze-dried wheat straw material obtained from the wheat straw cultures. FTIR analysis was performed using Spectrum One (Perkin-Elmer) equipped with a diamond top plate accessory that enables analysis of powdered materials. Dried and ground wheat straw taken from the culture flasks was applied directly to the diamond and pressed using a pressure arm. Spectra were acquired for the wavenumber range 850–1850 cm^−1^ at the spectral resolution 4 cm^−1^ and 256 scans were taken for each spectrum. Three spectra were obtained for each sample and the triplicate averaged spectrum was used to perform principal component analysis (PCA) using The Unscrambler software (CAMO). Spectra were peak normalized and were linear corrected before performing PCA. Nuclear magnetic resonance NMR experiments were performed using a Bruker Avance 400 spectrometer, equipped with a Bruker 4-mm MAS double-resonance probe head, at ^13^C and ^1^H frequencies of 100.5 and 400.0 MHz, respectively. The spinning frequencies (at 14 kHz) were controlled by a pneumatic system that ensures a rotation stability higher than ~ 2 Hz. Typical π/2 pulse lengths of 4.2 and 3.0 µs were applied for ^13^C and ^1^H, respectively. Proton decoupling field strength of γB_1_/2π = 75 kHz was used. ^13^C quantitative spectra were measured by using the multiple-CP (MultiCP) method described by Johnson and Schmidt-Rohr [60]. A total of nine CP blocks were implemented with 1 ms and RF amplitude increment (90–100%), while the last CP before acquisition was executed with 0.8 ms and the same amplitude increment. The recycle delay was 2 s and the duration of the repolarization period *t*_z_ was 0.9 s [60]. To aid in the analyses of the ssNMR results, the multivariate curve resolution (MCR) procedure [61] was carried out using the software The Unscrambler X^®^ v10.4.1 (CAMO Software AS). The basic goals of MCR are as follows: the determination of the number of components co-existing in the chemical system; the extraction of the pure spectra of the components (qualitative analysis); and the extraction of the concentration profiles of the components (quantitative analysis). This analysis is preceded by principal component analysis (PCA) to estimate the number of components in the mixture. After this, the rotation of the PC is calculated without orthonormality constrains (in this way it will have infinite solutions). To solve this, new constrains are adopted (e.g. non-negative concentrations and non-negative spectra). In this way, when the goals of MCR are achieved, it is possible to unravel the “true” underlying sources of data variation, and then the results with physical meaning are easily interpretable.

### Enzyme activity

Release of reducing sugars was determined with Lever assay [62]. Filtered sterilized (0.22-μm PES filter unit) culture supernatant was incubated with 1% (w/v) of the appropriate polysaccharide substrate (in 50 mM sodium phosphate buffer, pH 6.5) at 37 °C for different time intervals. The reaction was stopped by adding *p*-hydrobenzoic acid (PAHBAH) reagent followed by heating at 70 °C for 10 min. The release of reducing sugars was determined in a microtitre plate Tecan Sunrise plate reader at 415 nm. Dilutions of a stock solution of 1 mg/ml of glucose or xylose were assayed to obtain a standard curve.

### Data analysis

Nucleotide sequences for contigs identified by Mascot database searching as having matches to observed proteins were retrieved from the metatranscriptomic databases using Blast-2.2.30 + Standalone. EMBOSS [63] application getorf was used to generate all possible open reading frames (ORFs) from these matched contigs, defined as any region > 300 bases between a start (ATG) and STOP codon. These ORF libraries were converted into amino acid sequences and then used as the databases for a second round of searches with the original tandem mass spectral data. Results were filtered through Mascot Percolator and adjusted to accept only peptides with an expect score of 0.05 or lower. An estimation of relative protein abundance was performed as described by Ishihama [64]. Molar percentage values were calculated by normalizing the Mascot derived emPAI values against the sum of all emPAI values for each sample.

Protein sequences from ORFs identified as being present in the metasecretomes were annotated using BLASTP searching against the non-redundant NCBI database with an E-value threshold of 1 × 10^−20^. The BLASTX xml output files were used to taxonomically assign metasecretome in MEGAN 5.10.5 [65] and compare taxonomic distribution between time points and proteome fraction. Additionally, protein sequences were annotated using dbCAN [66] to identify likely carbohydrate-active domains (if alignment length > 80 aa, E-value < 1 × 10^−5^ was used, otherwise E-value < 1 × 10^−3^ was applied). Subcellular localisation was predicted using TMHMM v. 2.0 [67] and SignalP v. 4.1 [68] servers with default cut off values. The protein sequences were functionally annotated using WebMGA server and RPSBLAST program on COG database [69]. Venn diagrams were constructed using package VennDiagram v. 1.6.9 in R. Hierarchical clustering was performed using package ecodist v. 1.2.9 [70] or vegan v. 2.2-1 in R to evaluate the relationship between samples based on the OTU counts, protein molar abundance and expression pattern. Heatmaps were constructed using ggplot (v. 3.0.1) package in R.

## Additional files

**Additional file 1: Table S1.** Number of sequences, OTUs and alpha indices from Ion Torrent sequencing of 16S and 18S amplicons of samples. **Table S2.** Number of raw, rRNA and quality filtered sequences from the RNA-seq metatranscriptomics. **Table S3.** Number of unassigned and assigned spectra through analysis of biotin-labelled (BF) and supernatant (SNT) fractions of the wheat straw cultures.

**Additional file 2: Figure S1.** Morphological changes of the wheat straw biomass collected from weekly time points. **Figure S2.** Rarefaction analysis of prokaryotic (**a**) and eukaryotic (**b**) community from the wheat straw cultures based on rRNA amplicon sequencing. **Figure S3.** Overview of the wheat straw degrading community metatranscriptome. **Figure S4.** Overview of the metasecretome of wheat straw degrading community. **Figure S5.** Comparison of Clusters of Orthologous Groups (COGs) in the metasecretome (MP) and metatranscriptome (MT).

**Additional file 3.** Overview of proteins detected in the metasecretome of the wheat straw compost-derived community.

## Abbreviations

FTIR: fourier transform infrared spectroscopy
PCA: principal component analysis
ssNMR: solid-state nuclear magnetic resonance
MT: metatranscriptome
LC–MS/MS: liquid chromatography-tandem mass spectrometry
OTU: operational taxonomic unit
COG: cluster of orthologous group
LPMO: lytic polysaccharide monooxygenase
CAZy: carbohydrate-active enzymes
GH: glycosyl hydrolase
AA: auxiliary activities
CBM: carbohydrate-binding module
GT: glycosyl transferase
PL: polysaccharide lyase
ORF: open reading frame
