# Supplementary material for: Defining functional diversity for lignocellulose degradation in a microbial community using multi-omics studies
Source: Biotechnol Biofuels. 2018 Jun 18;11:166. doi: 10.1186/s13068-018-1164-2 (PMC6004670; doi:10.1186/s13068-018-1164-2)

**Supplementary Tables**

# Defining functional diversity for lignocellulose degradation in a microbial community using multi-omics studies.

Anna M. Alessi^1^, Susannah M. Bird^1^, Nicola C. Oates^1^, Yi Li^1^, Adam A. Dowle^2^, Etelvino Henrique Novotny^3^, Eduardo R deAzevedo^4^, Joseph P. Bennett^1^, Igor Polikarpov^4^, J. Peter W. Young^5^, Simon J. McQueen-Mason^1^, Neil C. Bruce^1^*

^1^Centre for Novel Agricultural Products, Department of Biology, University of York, York, YO10 5DD, UK

^2^Bioscience Technology Facility, Department of Biology, University of York, York, YO10 5DD, UK

^3^Embrapa Solos, Rio de Janeiro-RJ, Brazil.

^4^Grupo de Biotecnologia Molecular, Instituto de Física de São Carlos, Universidade de São Paulo, São Carlos-SP, Brazil

^5^Department of Biology, University of York, York, YO10 5DD, UK

*Correspondence should be addressed to N.C.B ([neil.bruce@york.ac.uk](mailto:neil.bruce@york.ac.uk))

**Content**

Table S1

Table S2

Table S3

**Table S1**. Number of sequences, OTUs and alpha indices from Ion Torrent sequencing of 16S and 18S amplicons of samples obtained from weekly time points and inoculum.


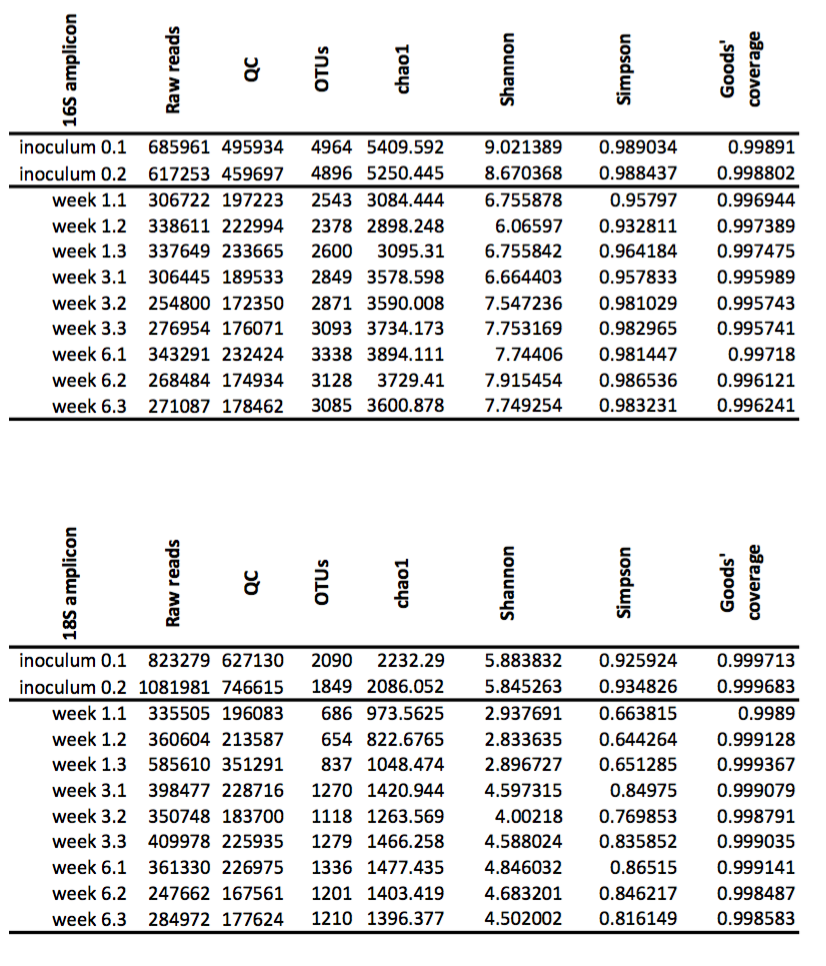


**Table S2**. Number of raw, rRNA and quality filtered sequences from the RNA-seq metatranscriptomics


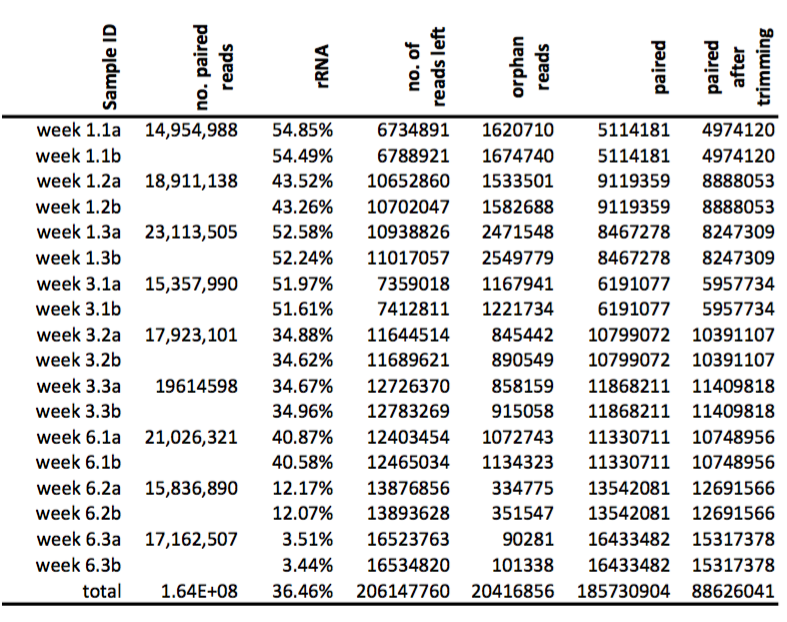


**Table S3**. Number of unassigned and assigned spectra through analysis of biotin-labelled (BF) and supernatant (SNT) fractions of the wheat straw cultures.

a - spectra searched using MASCOT engine and metatranscriptome database

b - spectra searched using MASCOT engine and ORF database (6,760 sequences; 1,868,362 residues)


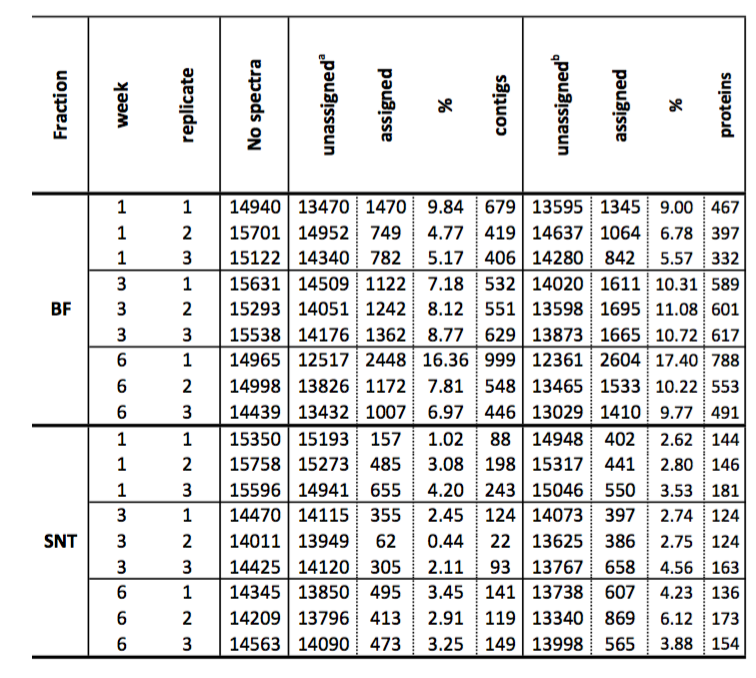

Supplement: Supplementary file 1 — Additional file 1: Table S1. Number of sequences, OTUs and alpha indices from Ion Torrent sequencing of 16S and 18S amplicons of samples. Table S2. Number of raw, rRNA and quality filtered sequences from the RNA-seq metatranscriptomics. Table S3. Number of unassigned and assigned spectra through analysis of biotin-labelled (BF) and supernatant (SNT) fractions of the wheat straw cultures. [file 13068_2018_1164_MOESM1_ESM.docx]
