# Supplementary material for: Defining functional diversity for lignocellulose degradation in a microbial community using multi-omics studies
Source: Biotechnol Biofuels. 2018 Jun 18;11:166. doi: 10.1186/s13068-018-1164-2 (PMC6004670; doi:10.1186/s13068-018-1164-2)
Supplement: Supplementary file 2 — Additional file 2: Figure S1. Morphological changes of the wheat straw biomass collected from weekly time points. Figure S2. Rarefaction analysis of prokaryotic (a) and eukaryotic (b) community from the wheat straw cultures based on rRNA amplicon sequencing. Figure S3. Overview of the wheat straw degrading community metatranscriptome. Figure S4. Overview of the metasecretome of wheat straw degrading community. Figure S5. Comparison of Clusters of Orthologous Groups (COGs) in the metasecretome (MP) and metatranscriptome (MT). [file 13068_2018_1164_MOESM2_ESM.docx]

**Supplementary Figures**

# Defining functional diversity for lignocellulose degradation in a microbial community using multi-omics studies.

Anna M. Alessi^1^, Susannah M. Bird^1^, Nicola C. Oates^1^, Yi Li^1^, Adam A. Dowle^2^, Etelvino Henrique Novotny^3^, Eduardo R deAzevedo^4^, Joseph P. Bennett^1^, Igor Polikarpov^4^, J. Peter W. Young^5^, Simon J. McQueen-Mason^1^, Neil C. Bruce^1^*

^1^Centre for Novel Agricultural Products, Department of Biology, University of York, York, YO10 5DD, UK

^2^Bioscience Technology Facility, Department of Biology, University of York, York, YO10 5DD, UK

^3^Embrapa Solos, Rio de Janeiro-RJ, Brazil.

^4^Grupo de Biotecnologia Molecular, Instituto de Física de São Carlos, Universidade de São Paulo, São Carlos-SP, Brazil

^5^Department of Biology, University of York, York, YO10 5DD, UK

*Correspondence should be addressed to N.C.B ([neil.bruce@york.ac.uk](mailto:neil.bruce@york.ac.uk))

**
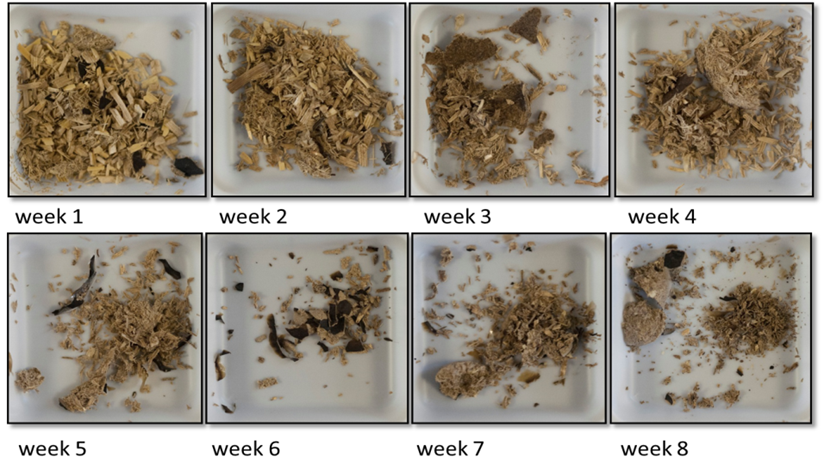
**

**Figure S1**. Morphological changes of the wheat straw biomass collected from a weekly time points. The darkening and size reduction was noted during the experiment.

**Figure S2**. Rarefaction analysis of prokaryotic (a) and eukaryotic (b) community from the wheat straw cultures based on rRNA amplicon sequencing.

Number of observed species was identified in the inoculum, week 1, 3 and 6. Each curve illustrates average observed species obtained from two iterations of rarefied subset of all sequences (on x-axis). Error bars were calculated for biological replicates of inoculum (n=2), week 1, 3 and 6 (n=3).

**Figure S3**. Overview of the wheat straw degrading community metatranscriptome.

**a** Venn diagram of unique and shared transcripts in samples collected at week 1, 3 and 6. **b** Hierarchical clustering analysis of metatranscriptomic normalised ESTs of the wheat straw degrading cultures. Analysis was performed in R with Euclidean distance and complete method. Colouring: red = week 1, dark teal = week 3, dark blue = week 6.


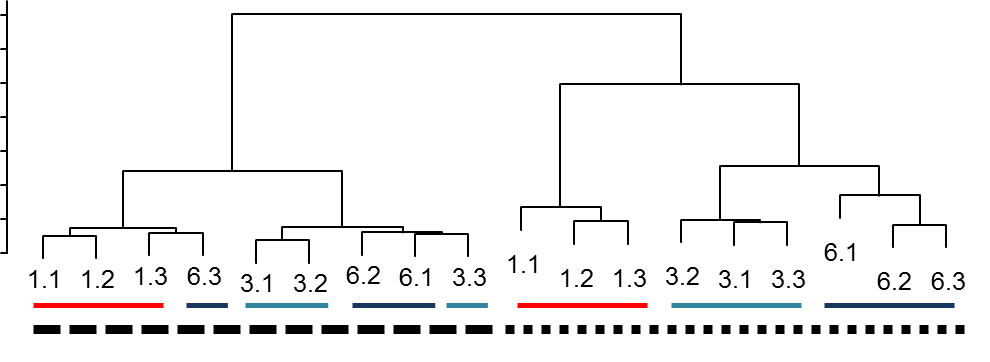


**Figure S4.** Overview on metasecretome of wheat straw degrading community.

Hierarchical clustering analysis of proteins from wheat straw degrading cultures identified in biotin-labelled (dashed line) and supernatant fraction (dash-dotted line). Analysis was performed in R with Bray-Curtis distance and ward.2 method. Colouring: red = week 1, dark teal = week 3, dark blue = week 6.


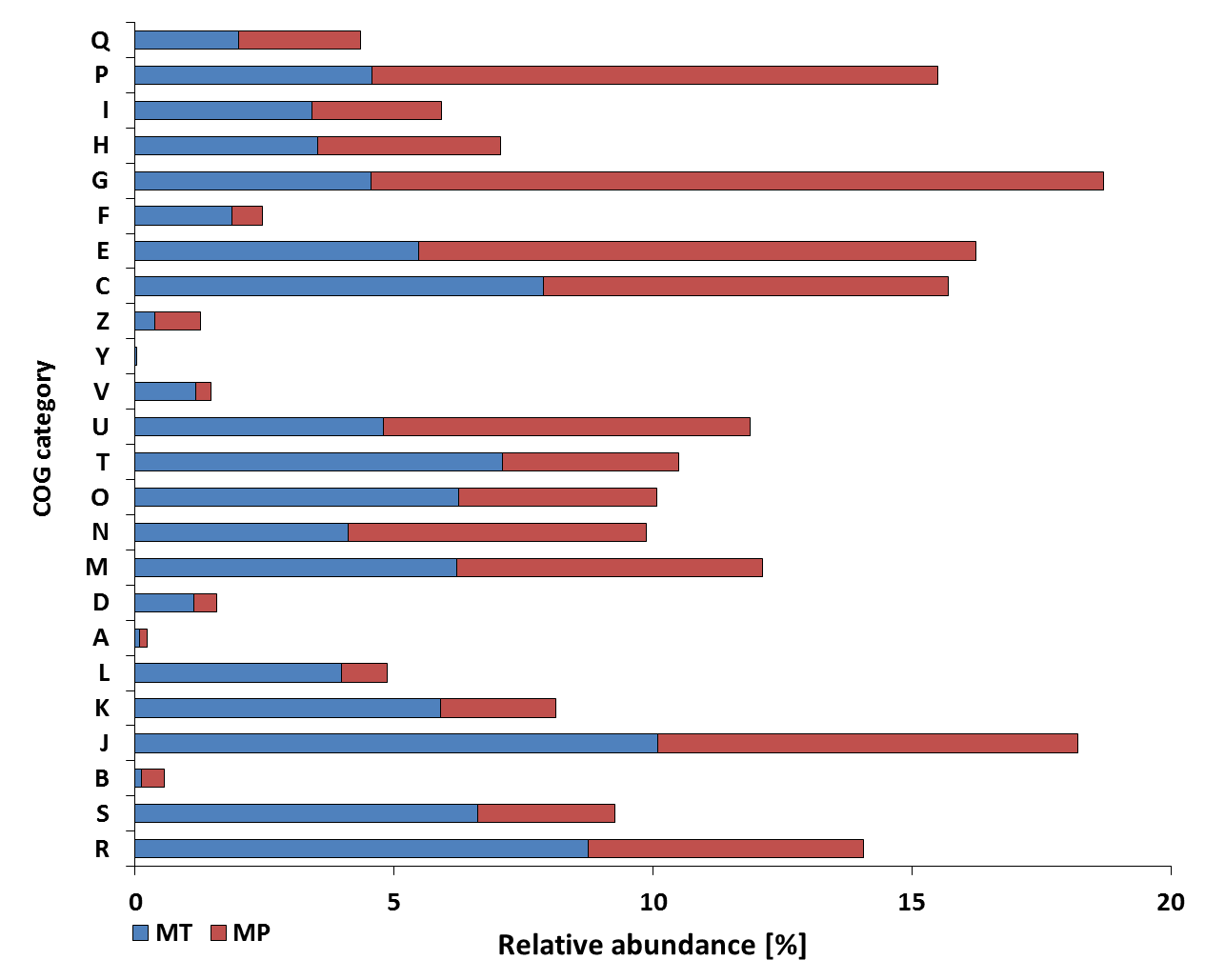


**Figure S5**. Comparison of Clusters of Orthologous Groups (COGs) in the metasecretome (MP) and metatranscriptome (MT).

The predicted genes from the MT and MP contigs were mapped to different COGs using WebMGA server and RPSBLAST program. [D] Cell cycle control, cell division, chromosome partitioning [M] Cell wall/membrane/envelope biogenesis [N] Cell motility [O] Post-translational modification, protein turnover, and chaperones [T] Signal transduction mechanisms [U] Intracellular trafficking, secretion, and vesicular transport [V] Defense mechanisms [W] Extracellular structures [Y] Nuclear structure [Z] Cytoskeleton [A] RNA processing and modification [B] Chromatin structure and dynamics [J] Translation, ribosomal structure and biogenesis [K] Transcription [L] Replication, recombination and repair [C] Energy production and conversion [E] Amino acid transport and metabolism [F] Nucleotide transport and metabolism [G] Carbohydrate transport and metabolism [H] Coenzyme transport and metabolism [I] Lipid transport and metabolism [P] Inorganic ion transport and metabolism [Q] Secondary metabolites biosynthesis, transport, and catabolism [R] General function prediction only [S] Function unknown
